# Public perception towards marriage involving individuals with bipolar disorder and its relationship with affirming attitudes among general population

**DOI:** 10.1192/j.eurpsy.2025.1090

**Published:** 2025-08-26

**Authors:** Y. Trabelsi, B. N. Saguem, N. Jaafar

**Affiliations:** 1 Psychiatry Department, Farhat Hached Hospital, sousse, Tunisia

## Abstract

**Introduction:**

Public perceptions regarding marriage in individuals with bipolar disorder are often influenced by societal stigma and misconceptions. These views can shape attitudes towards their suitability for long-term relationships. Misunderstandings about mental illness often create barriers to social acceptance.

**Objectives:**

This study aims to explore the general population’s perceptions of marriage involving individuals with bipolar disorder and assess how these views relate to broader affirming attitudes towards mental health.

**Methods:**

Across sectional study was conducted via an online formulary shared on social media. It included a detailed description of clinical symptoms and outcomes of bipolar disorder along with 13 questions assessing the perception of the participants about marrying an individual with bipolar disorder. A battery for measurement of affirming attitudes was used, it comprised 3 self-report measures: The Empowerment scale(ES), the Recovery scale(RS) and the self-discrimination scale(SDS).

**Results:**

A total of 304 participants were included, with the majority aged between 20 and 30 years, 71 participant indicated that they were living with someone diagnosed with a psychiatric disorder. Results show that opinions on marriage for individuals with bipolar disorder are mixed. While 50.3% believe such individuals can marry, there is significant doubt about marriage improving symptoms with 63.5% disagreeing that it helps, and 61.8% rejecting the idea of marriage as a cure. Moreover, 53.0% believe that marriage could worsen symptoms. 55.9% would not personally agree to marry someone with this condition. Similarly, 58.6% would not approve of a relative marrying someone with bipolar disorder. However, despite these concerns, 80.6% agree that people with bipolar disorder have the right to make their own decisions concerning marriage.

Additionally, participants who expressed a refusal to marry someone with bipolar disorder (p<10^-3) had significantly higher RS scores. Moreover, participants who believed that individuals with bipolar disorder are dangerous to their partners exhibited significantly higher ES scores (p=0.036). Furthermore, a significant result was observed among participants who disagreed that individuals with bipolar disorder are capable of making decisions regarding marriage, these individuals demonstrated elevated scores on all three scales: RS (p<10^-3), ES (p=0.02), and SDS (p=0.01).

**Conclusions:**

This study highlights the ongoing challenge of societal stigma towards individuals with bipolar disorder, particularly regarding marriage. While there are encouraging signs of changing attitudes among certain demographic groups, broader efforts are needed to foster a more inclusive and supportive societal perspective on this topic.

**Disclosure of Interest:**

None Declared

